# Globotrioasylsphingosine Levels and Optical Coherence Tomography Angiography in Fabry Disease Patients

**DOI:** 10.3390/jcm10051093

**Published:** 2021-03-05

**Authors:** Maximilian Robert Justus Wiest, Mario Damiano Toro, Albina Nowak, Joel Baur, Katrin Fasler, Timothy Hamann, Mayss Al-Sheikh, Sandrine Anne Zweifel

**Affiliations:** 1 Department of Ophthalmology, University Hospital Zurich, University of Zurich, 8091 Zurich, Switzerland; maximilian.wiest@usz.ch (M.R.J.W.); toro.mario@email.it (M.D.T.); mail@joelbaur.ch (J.B.); katrin.fasler@usz.ch (K.F.); timothy.hamann@usz.ch (T.H.); mayss.al-sheikh@usz.ch (M.A.-S.); 2 Faculty of Medical Sciences, Collegium Medicum, Cardinal Stefan Wyszyński University, 01815 Warsaw, Poland; 3 Department of Endocrinology and Clinical Nutrition, University Hospital Zurich, University of Zurich, 8091 Zurich, Switzerland; albina.nowak@usz.ch; 4 Department of Internal Medicine, Psychiatry University Clinic Zurich, 8091 Zurich, Switzerland

**Keywords:** fabry disease, globotrioasylsphingosine, optical coherence tomography angiography, lysosomal storage disorder, vessel density, vessel length density

## Abstract

Background: To date, there are no studies associating the dried blood spot (DBS) levels of globotrioasylsphingosine (lysoGb3) with quantitative optical coherence tomography angiography (OCTA) parameters in Fabry disease (FD) patients. Here, we aimed to investigate the association between OCTA vessel density (VD), vessel length density (VLD) with DBS lysoGb3. Methods: A retrospective, single center analysis of all consecutive FD patients enrolled at the Department of Ophthalmology of the University Hospital of Zurich from 1 December 2017 to 9 September 2020. An association between VD and VLD detected by OCTA and lysoGb3 was investigated using a linear mixed model. Results: A total of 57 FD patients (23 male, 34 female; 109 eyes) were included. Forty-one patients suffered from the classic phenotype and 16 from the later-onset phenotype. LysoGb3 inversely correlated with VD and VLD in both the superficial (VD: *p* = 0.034; VLD: *p* = 0.02) and deep capillary plexus (VD: *p* = 0.017; VLD: *p* = 0.018) in the overall FD cohort. Conclusions: Our study shows an association between lysoGb3 and OCTA VD and VLD. This supports the hypothesis that quantitative OCTA parameters might be useful as diagnostic biomarkers for evaluating systemic involvement in FD, and possibly other diseases.

## 1. Introduction

Fabry disease (FD) is a rare lysosomal storage disorder in which globotrioasylsphingosine (Gb3) accumulates in fluids and lysosomes of patients [1]. Mutations in the α-galactosidase gene (GLA) cause a decrease in overall activity of the enzyme α-galactosidase (α-Gal A) [2], which leads to accumulation of the aforementioned substrate in tissues and organs including the heart, kidney, the nervous system, the skin, vascular endothelium and the eye and causes progressive organ dysfunction^3^. Renal failure, cardiovascular disease and premature strokes are amongst the most common and severe fatal complications [1,2,3,4,5].

As an X-linked disease, FD predominantly affects males, the incidence being 1 in 40,000 to 117,000 live births for males [6,7]. Two phenotypes exist: the classic male phenotype with very low α-Gal A activity can be distinguished from the later-onset male phenotype with significant residual α-Gal A activity by manifestation of early symptoms such as acroparesthesias, abdominal cramping and hypohydrosis, while the later-onset patients typically develop hypertrophic cardiomyopathy or chronic kidney disease during adulthood [2,8,9]. In heterozygous females, due to random X-chromosomal inactivation, the phenotypes are more heterogenous, with a disease activity ranging from very mild to rather severe [10].

Ocular manifestations usually occur in the classic phenotype sometimes contributing to an early diagnosis of the disease [11]. The most common, and often key in early diagnosis of FD, is a vortex-like opacity found in the cornea, cornea verticillata (CV) [12,13,14]. Another specific finding, in fact, the only pathognomonic ocular finding, is a spoke-like posterior cataract which can be observed in 9.8% of females and 21.9% of males [15,16]. FD also manifests in the retina. Previous studies report an increased tortuosity of retinal vessels in patients suffering predominantly from the classic phenotype [9].

In the last decades, intravenous enzyme replacement therapy (ERT) with agalsidase alfa (Replagal^®^, Shire, Lexington, MA, USA) or agalsidase beta (Fabrazyme^®^, Sanofi Genzyme, Cambridge, MA, USA) has become the gold standard therapy of FD [17,18,19]. Recently, pharmacological chaperone therapy has been approved for amenable mutations [20].Under ERT, LysoGb3 levels have been reported to decrease, objective clinical parameters such as cerebral blood flow and symptoms to improve [21,22]. Better results have been reported when ERT was initiated before fibrosis and permanent organ damage has occurred [21].

Optical coherence tomography angiography (OCTA) is a non-invasive infrared-based imaging technique that has been developed in recent years [23]. It is capable of delivering structural and angiographic analysis without invasive measures such as intravenous dye injection [24]. OCTA is able to provide quantitative microvascular information such as vessel density (VD) and vessel length density (VLD) [25,26]. Additionally, OCTA allows a clear and detailed visualization of retinal and choroidal microvasculature, and it is useful either for diagnostic purposes or for guiding treatment, and monitoring different retinal diseases [27]. Past publications have shown that differences in quantitative OCTA parameters, such as VD and VLD in between patients suffering from FD and healthy controls are detectable [28,29,30]. Furthermore, a recent publication from Cennamo et al. [31] from December 2020 has demonstrated that quantitative OCTA parameters inversely correlate with echocardiographic parameters of myocardial disease and, thus, might be used as surrogate biomarkers for systemic involvement and progression in and of FD. Indeed, OCTA, identifying subclinical microvascular modifications [24], could potentially be used as a “multidisciplinary” instrument, not exclusively by ophthalmologists, for the early diagnosis and management of several systemic diseases [32].

To our knowledge to date, there are no studies associating the dried blood spot (DBS) levels of globotrioasylceramide (lysoGb3) with quantitative OCTA parameters. In this study, we aimed to investigate the association between OCTA VD, VLD of the superficial (SCP) and deep retinal capillary plexus (DCP) with DBS lysoGb3.

## 2. Materials and Methods

This is a single-center, retrospective analysis of a cohort of consecutive patients with genetically proven diagnosis of FD who underwent annual ophthalmological checkup at the medical retina unit of the Department of Ophthalmology, University Hospital of Zurich, Switzerland from 1 December 2017 to 9 September 2020. The institutional review board approved this study (Cantonal Ethics Committee, Canton of Zurich, BASEC-No. 2019-02043) and the tenets of the Declaration of Helsinki were followed. A written informed consent for the processing of personal data was obtained from each patient.

Inclusion criteria were as follows: genetically confirmed diagnosis of FD, minimal age of 16 years of age. Exclusion criteria were: evidence of ocular and systemic diseases unrelated to FD, current or previous macular and retinal vascular diseases, diagnosis of glaucoma, congenital eye disease, high myopia (>4 dioptres), significant lens opacification and reduced-quality OCTA images (signal strength of 7/10 or lower).

All patients underwent a blood test and an ophthalmological examination with best corrected visual acuity (BCVA), intraocular pressure (IOP) measurement, slit lamp examination of the anterior segment and biomicroscopy of the fundus, ultrawide-field scanning laser ophthalmoscopy (Optomap, Optos, Marlborough, MA, USA), spectral-domain optical coherence tomography (SD-OCT) and swept-source OCTA.

LysoGb3 concentrations were measured in DBS using highly sensitive electrospray ionization liquid chromatography tandem mass spectrometry (ESI LC-MS/MS), modified according to Gold et al. [33] as previously described by Nowak et al. [34]. A 7-point serum calibrator and an internal standard for LysoGb3 quantification (covering the analytic range from 0 to 120 ng/mL; lower limit of quantification: 0.2 ng/mL), and three calibrator levels (3, 30 and 100 ng/mL) for quality control were used (ARCHIMED Life Science GmbH, Vienna, Austria; www.archimedlife.com accessed on 1 March 2021).

BCVA was assessed using glasses with refractory values obtained using an autorefractometer (NT-530/510^®^, NIDEK Inc., San Jose, CA, USA) values were then converted to Early Treatment Diabetic Retinopathy Study (ETDRS) number values [35]. Intraocular pressure measurements were acquired using the same device.

OCTA was performed using the swept-source PLEX Elite 9000 device, software version 2.0.1.47652 (Carl Zeiss Meditec Inc., Dublin, CA, USA). 3 mm × 3 mm scans centered on the fovea were acquired by a well-trained, certified ophthalmologist. Scans with a signal strength of 8 out of 10 or higher were included, as signal strength has been shown to influence quantitative measurements in OCTA [36]. Additionally, scans with incorrect centration, bad focus, motion or errors in projection artifact removal were excluded.

Quantitative parameters of the 3 × 3 mm cube scans were automatically generated using layer segmentation produced by the instrument software and prototype analysis vascular density quantification software (Macular Density v.0.7.1, ARI Network Hub, Carl Zeiss Meditec Inc., Dublin, CA, USA) supplied by the manufacturer. Output values are vascular density metrics previously used in the literature, such as VD, which represents the fraction of a measured region with perfusion signal and VLD, which represents the total length of perfused vasculature in a measured area, given in millimeters of perfused vasculature per square millimeter of area, or inverse millimeters (mm^−1^) [37,38]. The analyzed region of interest was the innermost ring of the ETDRS grid (inner ring, iR), with an inner diameter of 1 mm and an outer diameter of 3 mm, centered on the fovea [35].

Data such as age at examination date, gender, FD phenotype, BCVA, IOP, as well as the presence or absence of FD associated ophthalmological findings, as well as the lysoGb3 concentration from DBS samples and systemic involvement were acquired from electronic health reports and used for statistical analysis.

### Statistical Analysis

Statistical analysis was performed using SPSS software (version 26, IBM Corporation, Armonk, NY, USA). Clinical parameters and OCTA data were displayed using descriptive statistics. To test whether lysoGb3 had an influence on OCTA parameters, a linear mixed model was used. This allows for inclusion of both eyes from patients, wherever both were available by correcting for multiple measurements (right eye, left eye, repeated covariance setting: compound symmetry).

Analysis of the influence of lysoGb3 on OCTA parameters in the overall cohort was performed. The influence of laterality, sex and age were accounted for by including laterality and sex as cofactors and age as covariance. In case of a *p*-value of lower than 0.05 for the type III test of fixed effect (F-test), the estimates of fixed effect of lysoGb3 on the OCTA parameter were reviewed in detail. The aforementioned analysis was performed for VD in the SCP, VD in the DCP, VLD in the SCP and VLD in the DCP, for parameters gathered from the ETDRS grid inner ring area.

The statistical analysis was repeated for the following subgroups: male patients, female patients, classic phenotype patients and later-onset patients. Data for the overall cohort and the male and female subgroups were plotted as scatterplots with fit lines. For easier viewability, lysoGb3 values underwent natural log transformation (Ln.lysoGb3).

## 3. Results

Out of the 63 patients identified, 6 had to be excluded because no OCTA imaging of sufficient quality was available. Of the remaining 57 patients with 114 eyes, 5 eyes had to be excluded because of incorrect centering or bad focus (3 and 2, respectively). Among 109 eyes, one DCP segmentation had to be excluded due to segmentation failure.

A total of 57 (34 female, 23 male) genetically proven FD patients with 109 (57 right, 52 left) eyes and 109 SCP and 108 DCP segmentations were included for analysis. Forty-one patients suffered from the classic phenotype (27 female, 14 male) and 16 from the later-onset phenotype (7 female, 9 male). Of all the patients, 40 (22 male, 18 female, 70.2% overall) were under ERT. Mean age was 43.4 (±15.3) years and mean lysoGb3 concentration was 16.7(±21.03; min. 1.5; max. 86.7) ng/mL.

Cornea verticillata was observed in 74 (67.9%) and retinal vessel tortuosity in 52 (47.7%) eyes. None displayed a spoke-like posterior cataract. Mean BCVA was 1.04(±0.23) and mean IOP 14.8(±2.9) mmHg. A summary of demographics, genetical and clinical characteristics are shown in Table 1, Table 2 and Table 3.

Mean VD of the SCP was 0.382 ± 0.023 for the inner ring area. For the DCP, mean VD was 0.275 ± 0.049. The mean VLD of the SCP was 17.183 mm^−1^ ± 1.262 mm^−1^. In the DCP, VLD measured was lower with 12.747 mm^−1^ ± 2.248 mm^−1^ for the inner ring area.

Detailed information about means and standard deviations (SD) of OCTA parameters are shown in Table 4.

In the pooled FD group, lysoGb3 was identified as a statistically significant (*p* < 0.05) influence on VD and VLD in both, the SCP and DCP segmentations. The estimates of regression were −0.000338 (*p* = 0.034) for VD in SCP, −0.000714 (*p* = 0.020) for VD in DCP, −0.020157 (*p* = 0.17) for VLD in SCP and −0.032385 (*p* = 0.018) for VLD in DCP.

**Figure 1:**
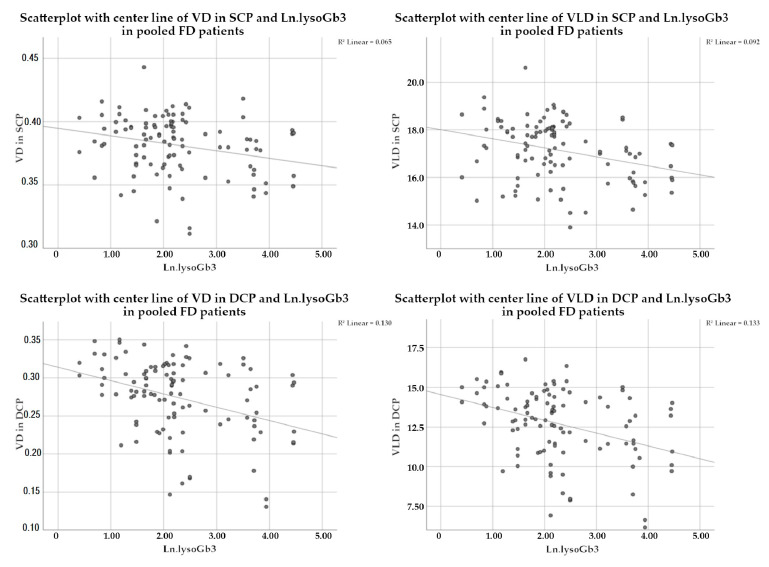
Scatterplots with centerline of VD and VLD in SCP and DCP of FD patients. In the upper left plot, the inverse association of VD in SCP with the logarithmized values of lysoGb3 (Ln.lysoGb3) is shown. The other plots show the inverse association for VLD in SCP (top right), VD in DCP (bottom left), and VLD in DCP (bottom right) with Ln.lysoGb3. Abbreviations: VD: vessel density; VLD: vessel length density; SCP: superficial capillary plexus; DCP: deep capillary plexus; FD: Fabry Disease; Ln.LysoGb3: logarithmized lysoGb3.

When repeating this analysis for the subgroup of male FD patients, we found that lysoGb3 showed a statistically significant influence for the VD and VLD in the SCP only with estimates of regression of −0.000349 (*p* = 0.014) for VD and −0.021716 (*p* = 0.005) for VLD. LysoGb3 did not appear as a statistically significant effect on VD and VLD in the DCP of the male subgroup. In the female subgroup as well as the subgroups of classic and later-onset phenotypes, no statistically significant influences of lysoGb3 were detected. A detailed view of *p*-values and estimates of regression with standard error (SE) are shown in Table 5 and a visualization of data for the pooled FD patients in Figure 1.

## 4. Discussion

In this study, we observed an inverse association between VD and VLD with lysoGb3 DBS levels in our cohort of genetically proven FD patients. We also show an inverse association of lysoGb3 with VD and VLD of the SCP of our male subgroup, but not in the DCP. However, in the other subgroups, we were unable to detect any statistically significant association.

The clinical presentation of FD can vary substantially, depending on the residual activity of α-galactosidase [2]. Clinical findings range from the initially mild, often non-system involving later-onset phenotype to the severe, classic phenotype with almost no remaining enzyme activity [2]. Different authors have reported associations of lysoGb3 levels to cardiac, renal [39], pulmonary changes [40], as well as neurological deficits [41,42]. Aerts et al. have postulated that elevated lysoGb3 concentrations play a key role in the pathogenesis of FD and lysoGb3 may be considered as the hallmark of FD [43]. It has been shown that inflammation and innate immune response in FD are caused by elevated lysoGb3. Increased exposure of dendritic Toll-like receptors to lysoGb3 can trigger overexpression of inflammatory molecules and tissue inflammation, which leads to fibrosis and ultimately organ dysfunction and/or failure [44,45,46,47,48,49,50]. LysoGb3 levels in DBS samples are highly correlated with plasma lysoGb3 [34] and are used as a diagnostic and predictive biomarker in the treatment of FD patients [40,51]. Indeed, Nowak et al. have demonstrated that lysoGb3 is a useful biomarker for the diagnosis of FD in heterozygotes and can assist in determining whether heterozygotes should be considered for treatment, despite normal α-GalA activity [52].

Previous studies have shown that OCT-A is able to detect microvascular retinal alterations and could be useful as a biomarker in the early diagnosis and follow-up of patients affected by different systemic diseases [32,37,53,54]. However, despite the progress in the diagnosis and the treatment of FD, evidence of correlations of OCTA parameters and systemic findings is low and there are only few studies highlighting such associations [31,55]. Cennamo et al. have shown that the VD of both the SCP and DCP inversely associate with different echocardiographic parameters (E/e’ ratio, systolic pulmonary arterial pressure, left atrial volume index and intraventricular septal thickness) [31]. In a recent publication, Lin et al. reported that serum creatinine and cystatine C are inversely associated with VD in a cohort of 26 patients with FD^55^. Our findings show that lysoGb3 seems to be inversely associated with both VD and VLD in the SCP and DCP. To our knowledge, lysoGb3 is the only FD-specific systemic biomarker for which associations with OCTA parameters have been observed.

Our results further support the hypothesis posed by Cennamo et al., that OCTA could represent a valid biomarker to predict systemic involvement in FD. Early detection of changes in OCTA parameters might be useful in identifying patients with subclinical systemic findings and, as has been outlined before, enable more effective ERT. This might be a key factor in preventing irreversible organ damage and development of more severe complications. In heterozygous females, α-Gal A activity can vary widely due to random X-chromosome inactivation [10] and female FD patients typically suffer from less and milder symptoms than male FD patients [52]. In accord with these findings by Nowak et al., we could only detect associations of lysoGb3 and OCTA parameters for VD and VLD in SCP in the male subgroup, not in the female subgroup. We suspect, this could be due to the milder manifestations in females with a lower mean and lower standard deviation of lysoGb3 in the female subgroup, even though the female subgroup was larger in our study.

The main limitations of this study are its retrospective nature, the limited sample size which is inevitable in rare diseases and the imbalance of lysoGb3 values in the male and female cohort.

## 5. Conclusions

In conclusion, our study shows an association between a FD specific parameter, lysoGb3, and quantitative OCTA parameters. The observed decrease of VD and VLD in patients with higher lysoGb3 supports the hypothesis that quantitative OCTA parameters might be useful as diagnostic biomarkers for evaluating systemic involvement in FD, and possibly other diseases.

Further prospective controlled trials with a large sample size and a long follow up to assess the dynamic changes of OCTA parameters with changing lysoGb3 values are highly demanded.

## Figures and Tables

**Table 1:** Demographics and clinical characteristics of Fabry disease (FD) patients.

		FD Patients *n* = 57
gender	male	23
	female	34
mean age (years) ± SD		43.4 ± 15.3
FD phenotype	classic	41
	later-onset	16
mean lysoGb3 (ng/mL) ± SD	overall	16.7 ± 21.03
	male	31.8 ± 27.03
	female	6.9 ± 3.52
enzyme replacement therapy *	yes	40
	no	17

*n*: number; lysoGb3: Globotriaosylceramide in dried blood spot samples; ng: nanograms; ml: milliliters; * agalsidase beta or ANN-agalsidase alfa.

**Table 2:** α-galactosidase gene (GLA) mutations observed in the pooled Fabry disease group and respective subgroups.

Mutation	Frequency				
	Pooled FD	Male *	Female *	Classic *	Later-Onset *
c.1033T > C	6	1	5	6	0
c.1055-1057dupCTA	2	1	1	2	0
c.114delCTT	1	0	1	1	0
c.1167dupT	2	0	2	2	0
c.1168insT	2	0	2	2	0
c.1196G > C	1	1	0	0	1
c.125T > C	3	1	2	3	0
c.337T > C	8	3	5	1	7
c.370-2A > G	1	1	0	1	0
c.514T > C	1	0	1	1	0
c.518C > T	1	1	0	1	0
c.559-560delAT	1	1	0	1	0
c.581C > T	7	2	5	7	0
c.613C > T	1	1	0	0	1
c.640-3C > G	1	0	1	1	0
c.644A > G	1	1	0	0	1
c.680G > A	1	0	1	1	0
c.704C > G	2	0	2	2	0
c.743-744delTA	2	1	1	2	0
c.744-745delTA	3	2	1	3	0
c.796G > T	1	1	0	1	0
c.827G > A	1	1	0	1	0
c.870G > C	1	0	1	0	1
c.899T > A	1	1	0	1	0
c.901C > T	1	2	1	1	0
c.902G > A	5	3	2	0	5

* referring to male, female, classic phenotype and later-onset phenotype subgroups.

**Table 3:** Ocular characteristics of study cohort.

		Eyes *n* = 109
laterality	right	57
	left	52
cornea verticillata	yes	74
	no	35
retinal vessel tortuosity	yes	52
	no	57
mean BCVA(EDTRS letters)		85.32 ± 4.92
mean IOP(mmHg)		14.8 ± 2.9

*n*: number; BCVA: best corrected visual acuity; ETDRS: Early Diabetic Treatment Retinopathy Study; IOP: intraocular pressure.

**Table 4:** Mean values of OCTA parameters.

OCTA Parameter		Mean	±SD
pooled FD group			
SCP	VD	0.382	0.023
	VLD	17.183	1.261
DCP	VD	0.275	0.049
	VLD	12.747	2.248
male FD subgroup			
SCP	VD	0.379	0.020
	VLD	16.955	1.113
DCP	VD	0.262	0.049
	VLD	12.137	2.207
female FD subgroup			
SCP	VD	0.384	0.026
	VLD	17.338	1.340
DCP	VD	0.283	0.047
	VLD	13.167	2.196
classic phenotype FD subgroup			
SCP	VD	0.379	0.025
	VLD	16.983	1.276
DCP	VD	0.269	0.051
	VLD	12.486	2.320
later-onset phenotype FD subgroup			
SCP	VD	0.391	0.017
	VLD	17.712	1.073
DCP	VD	0.289	0.043
	VLD	13.427	1.922

OCTA: Optical coherence tomography angiography; SD: standard deviation; FD: Fabry Disease; SCP: superficial capillary plexus; VD: vessel density; VLD: vessel length density; DCP: deep capillary plexus.

**Table 5:** Association of lysoGb3 and OCTA parameters.

OCTA Parameter		Regression Estimator	SE	*p*-Value *
pooled FD group				
SCP	VD	−0.000338	0.000155	0.034
	VLD	−0.020157	0.008128	0.017
DCP	VD	−0.000714	0.000297	0.020
	VLD	−0.032385	0.013286	0.018
male FD subgroup				
SCP	VD	−0.000349	0.000129	0.014
	VLD	−0.021716	0.006728	0.005
DCP	VD	−0.000655	0.000359	0.084
	VLD	−0.030220	0.015725	0.070
female FD subgroup				
SCP	VD	−0.000287	0.001157	0.806
	VLD	−0.018082	0.060351	0.766
DCP	VD	−0.001237	0.001745	0.484
	VLD	−0.053284	0.080509	0.513
classic phenotype FD subgroup				
SCP	VD	−0.000142	0.000234	0.547
	VLD	−0.009219	0.011554	0.430
DCP	VD	−0.000333	0.000413	0.426
	VLD	−0.014732	0.018550	0.432
later-onset phenotype FD subgroup				
SCP	VD	−0.001530	0.002049	0.471
	VLD	−0.051812	0.143124	0.725
DCP	VD	−0.004082	0.005504	0.474
	VLD	−0.128910	0.243568	0.607

OCTA: Optical coherence tomography angiography; SE: standard error; FD: Fabry’s Disease; SCP: superficial capillary plexus; VD: vessel density; VLD: vessel length density; DCP: deep capillary plexus; * Type III test for fixed effect; *p* was significant if *p* < 0.05.

## Data Availability

Data will be made available upon request to the corresponding author.
